# Postpartum Contraceptive Use Among US Medicaid Recipients

**DOI:** 10.1001/jamanetworkopen.2021.45175

**Published:** 2022-01-26

**Authors:** Maria I. Rodriguez, Thomas Meath, Kelsey Watson, Ashley Daly, Kyle Tracy, K. John McConnell

**Affiliations:** 1 Division of Complex Family Planning, Department of Obstetrics and Gynecology, Oregon Health & Science University, Portland; 2 Center for Health Systems Effectiveness, Oregon Health & Science University, Portland; 3 Department of Emergency Medicine, Oregon Health & Science University, Portland

## Abstract

This cohort study uses national Medicaid claims data to assess US state variation in effective contraceptive use 60 days post partum.

## Introduction

Improving access to postpartum contraception is a public health priority in the US.^1^ Postpartum contraception promotes health by preventing short interpregnancy intervals (<18 months from delivery), preterm birth, and associated health complications. More than half of all short interpregnancy interval pregnancies are unintended, and these pregnancies are concentrated among low-income individuals.^2^ Access to contraception is a critical dimension of high-quality family planning care.^3^

Medicaid, the largest single payer of obstetric and contraceptive services, provides an opportunity for improving access to postpartum contraception. However, access to postpartum coverage may vary across state Medicaid programs. Significant variations could point to systematic underuse of effective care and opportunities for state and federal policies to improve outcomes for Medicaid beneficiaries. Until recently, understanding these variations across states was hampered by the lack of national Medicaid claims data. We undertook a cohort study that used the 2016 Medicaid Transformed Medicaid Statistical Information System (T-MSIS) Analytic Files to assess state variation in effective contraceptive use 60 days post partum.

## Methods

This cohort study was conducted from January 1 to August 31, 2021. We determined the rate of postpartum contraceptive use by state or territory to categorize our findings. We excluded 6 states and territories with data assessed as “unusable” or “high concern” by using standardized quality assessments from the Center for Medicaid Services.^4^ Our data set included all live births to Medicaid recipients aged 15 to 44 years in 2016. The institutional review board at Oregon Health & Science University, Portland, approved the study. We received deidentified data from the Center for Medicaid Services; to protect patient confidentiality, a waiver for informed consent was given by the institutional review board at Oregon Health & Science University. We followed the Strengthening the Reporting of Observational Studies in Epidemiology (STROBE) reporting guideline for cohort studies.

We calculated the rate of individuals using an effective form of contraception (sterilization, contraceptive implants, intrauterine devices, injectables, oral pills, patch, ring, or diaphragm) within 60 days post partum, using the Office of Population Affairs specifications.^1^ We also determined the rate of individuals using long-acting reversible contraceptives (LARCs) within 60 days post partum.^1^ We used *International Statistical Classification of Diseases, Tenth Revision, Clinical Modification* diagnosis and procedure codes, National Drug Codes, and Healthcare Common Procedure Coding System codes to identify and classify contraceptive methods. We used R statistical software, version 4.0.3 R Project for Statistical Computing) to conduct our analyses.

## Results

Our final sample included 1 288 539 live births among 1 287 573 women receiving Medicaid,and living in 45 states and Washington, District of Columbia, in 2016. This cohort had a mean age of 27.1 (range, 15-44) years. Of these Medicaid enrollees, 34.2% were using an effective form of contraception within 60 days post partum. Rates varied substantially across states, with the lowest rate occurring in Utah (19.8%) and the highest rate occurring in Louisiana (43.9%) (Figure). Eight states had effective postpartum contraception rates at or above 40% and 4 states at or below 25%. Nationally, the proportion of women using LARCs within 60 days post partum was 9.3%, with a low of 2.7% in New Jersey and a high of 19.7% in Rhode Island.

**Figure.**
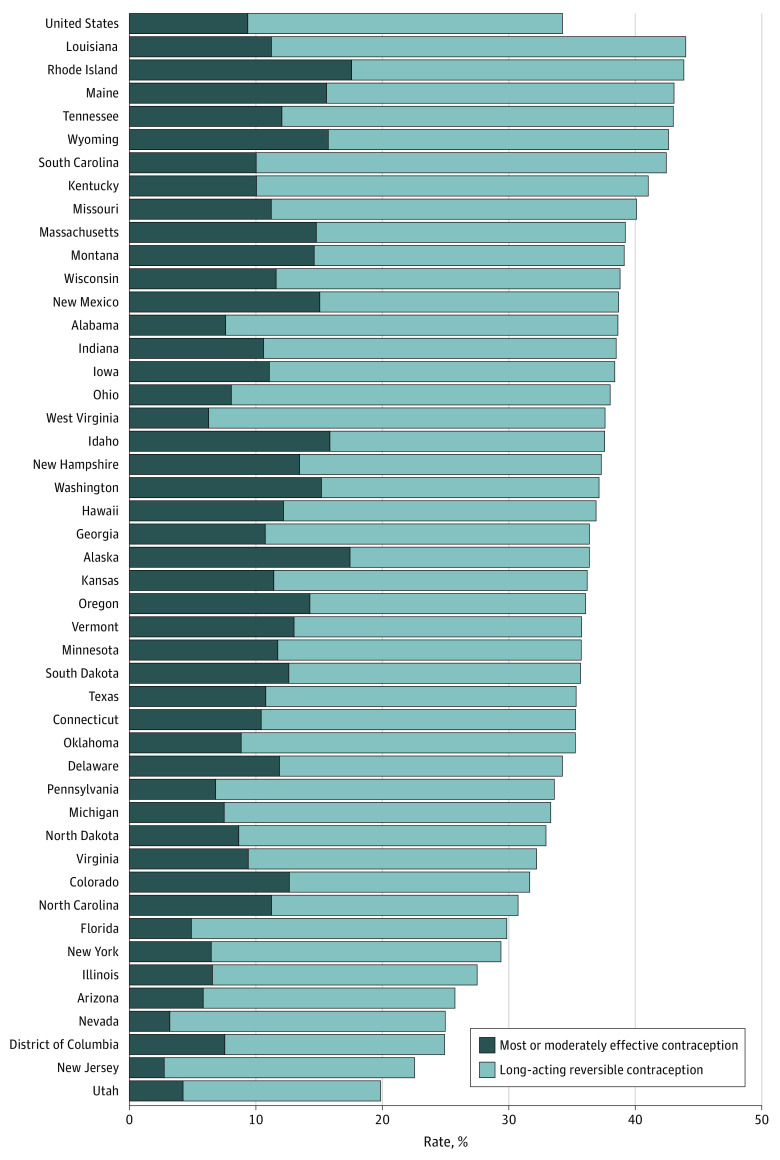
Rates of Effective Contraceptive and Long-Acting Reversible Contraceptive Use Within 60 Days Post Partum Among 1 287 887 US Medicaid Enrollees in 2016

## Discussion

The findings of this cohort study suggest a wide variation across states in effective postpartum contraception within Medicaid, with 8 states having rates at or above 40% and 4 states at or below 25%. Similar variations were observed in the use of postpartum LARCs. Within Medicaid, approximately 34% of women used an effective form of postpartum contraception in 2016, well below the national goal of 58.5%.^5^

Variability in the use of postpartum contraception may be associated with a variety of factors. There have been long-standing concerns that low reimbursement rates have made clinicians less willing to accept Medicaid patients. Administrative burdens and delays in billing may also limit clinicians’ willingness to provide some services. In addition, there may be inconsistencies across states in how postpartum contraception is covered or billed in the inpatient setting or the extent to which states monitor and incentivize high-quality contraceptive services. One option for states looking to increase the use of effective contraception is to provide financial incentives. In Oregon, effective contraception use increased by 11.5% 3 years after the Medicaid program introduced an incentive metric.^6^

Limitations of this study include its reliance on administrative data, which are subject to classification errors. To mitigate this potential source of bias, we excluded states with quality concerns by using standardized measures from the Data Quality Atlas.^4^ We do not capture patient-reported outcomes or preferences. In addition, our data are from 2016 and do not capture more recent trends in contraceptive use.

In conclusion, the findings of this cohort study showed substantial variation in postpartum contraceptive use nationally among Medicaid recipients. Such variation suggests opportunities to improve access to these services for millions of low-income women.
