# Error in Figure Key

**DOI:** 10.1001/jamanetworkopen.2022.3218

**Published:** 2022-02-24

In the Research Letter titled “Postpartum Contraceptive Use Among US Medicaid Recipients,”^1^ published January 26, 2022, the labels in the Figure’s color key were reversed. In the figure key, the dark blue color should indicate the rate of long-acting reversible contraception use within 60 days post partum and the light blue color should indicate the rate of most or moderately effective contraception use within 60 days post partum. This article has been corrected.^1^
